# Epidemiology, Public Health, and False-Positive Results: The Role of the Clinicians and Pathologists

**DOI:** 10.1289/ehp.1002047

**Published:** 2010-06

**Authors:** Francesco Cetta, Simona Benoni, Rosalia Zangari, Valentina Guercio, Massimo Monti

**Affiliations:** Department of Surgery, University of Siena, Siena, Italy, E-mail: cetta@unisi.it; Geriatric Institute PAT, Milan, Italy

We congratulate Blair et al. (2009) and Boffetta et al. (2008) for drawing proper attention to the rhetoric of false-positive results concerning environmental determinants and human health outcomes.

In particular, Boffetta et al. (2008) suggested that “users of epidemiological results outside the scientific community . . . should be aware of the fact that statistically significant or positive results are often false” and that “epidemiology is particularly prone to the generation of false-positive results.” Blair et al. correctly replied that not only could false-positive results be generated (2009) but also false-negative results. In particular, in a review of 39 highly cited (citation index > 1,000) randomized controlled trials that reported an original claim of an effect (Ioannidis 2005), only the results of 19 trials were replicated by subsequent studies. Therefore, caution should be applied in the communication of results to the media and the general public, because both tend to consider numbers and percentages as the “truth” and make their own speculations on data that are often based on inferences and weak associations.

However, the question of nonreproducibility of scientific results cannot be reduced to a mere controversy among epidemiologists—a controversy that should be limited strictly to them and treated only by improving statistical methods. It actually affects the basis of empirical knowledge, in particular when it involves biological and medical questions. When sensational new discoveries are counter to empirical observation, caution is mandatory. Biases may be detectable by epidemiologists, but there are other possible sources of basic errors concerning pathophysiologic mechanisms that are peculiar to each disease and that are unknown to statisticians, who apply the same methods to a wide variety of different conditions.

“Biological plausibility” is not enough. Individual susceptibility plays a role greater than previously supposed in the occurrence of clinical outcomes in the host due to environmental factors. The importance of susceptibility reflects a decreased relative role of pollutant concentration [i.e., intrinsic toxicity of xenobiotics (inhaled or ingested)] and reduces the applicability of certain models—based on dose and effect linearity—to no-threshold phenomena.

Proper selection of subgroups, which should be homogeneous not only for age and sex but also for pathophysiological relevance, is not an epidemiologist’s task but should be directed also by clinicians and pathologists. For example, lung cancer is still considered by epidemiologists as a single entity, but clinicians are aware that in addition to cancer occurring in an anthracotic lung, pulmonary cancers may also occur in nonanthracotic lungs; this is a different disease less likely to be dependent on air pollution. The knowledge of this fact will greatly affect population selection.

Finally, Boffetta’s criticism and plea for epistemological modesty is not only well timed but also necessary. In particular, in studies on health effects of environmental determinants, a common remark is that this field should never be the exclusive kingdom of environmentalists or epidemiologists; clinicians should play a vigorous role as scientists with direct experience. Therefore, a senior clinician having long-term experience with the disease of concern should always be involved in the design of the study and in reporting study results. Interdisciplinary control of research is not only a desirable option but a necessary measure to mitigate the sensational effect of new discoveries. This is true in particular when, despite statistical significance of observed differences, findings are counter to everyday clinical experience or they are not clearly adherent to—or a logical consequence of—strict criteria such as Koch’s postulates. Clinicians could also suggest the proper timing for large and expensive epidemiological trials, which should be performed exclusively when adequate metrics and reliable pathophysiologic causative mechanisms between determinants and outcomes have been established. Our view is that clinicians should be involved both in the study design and timing, so that interdisciplinary control of the study can be guaranteed from the beginning.
